# Diammonium Glycyrrhizinate Upregulates PGC-1α and Protects against Aβ_1–42_-Induced Neurotoxicity

**DOI:** 10.1371/journal.pone.0035823

**Published:** 2012-04-23

**Authors:** Xiaolei Zhu, Cong Chen, Dan Ye, Dening Guan, Lan Ye, Jiali Jin, Hui Zhao, Yanting Chen, Zhongyuan Wang, Xin Wang, Yun Xu

**Affiliations:** 1 Department of Neurology, Nanjing Drum Tower Hospital Clinical College of Traditional Chinese and Western Medicine, Nanjing University of Chinese Medicine, Nanjing, People's Republic of China; 2 Department of Neurology, Affiliated Drum Tower Hospital of Nanjing University Medical School, Nanjing, People's Republic of China; 3 The State Key Laboratory of Pharmaceutical Biotechnology, Nanjing University, Nanjing, People's Republic of China; 4 Jiangsu Key Laboratory for Molecular Medicine, Nanjing, People's Republic of China; Case Western Reserve University, United States of America

## Abstract

Mitochondrial dysfunction is a hallmark of beta-amyloid (Aβ)-induced neurotoxicity in Alzheimer's disease (AD), and is considered an early event in AD pathology. Diammonium glycyrrhizinate (DG), the salt form of Glycyrrhizin, is known for its anti-inflammatory effects, resistance to biologic oxidation and membranous protection. In the present study, the neuroprotective effects of DG on Aβ_1–42_-induced toxicity and its potential mechanisms in primary cortical neurons were investigated. Exposure of neurons to 2 µM Aβ_1–42_ resulted in significant viability loss and cell apoptosis. Accumulation of reactive oxygen species (ROS), decreased mitochondrial membrane potential, and activation of caspase-9 and caspase-3 were also observed after Aβ_1–42_ exposure. All these effects induced by Aβ_1–42_ were markedly reversed by DG treatment. In addition, DG could alleviate lipid peroxidation and partially restore the mitochondrial function in Aβ_1–42_-induced AD mice. DG also significantly increased the PGC-1α expression *in vivo* and *in vitro*, while knocking down PGC-1α partially blocked the protective effects, which indicated that PGC-1α contributed to the neuroprotective effects of DG. Furthermore, DG significantly decreased the escape latency and search distance and increased the target crossing times of Aβ_1–42_-induced AD mice in the Morris water maze test. Therefore, these results demonstrated that DG could attenuate Aβ_1–42_-induced neuronal injury by preventing mitochondrial dysfunction and oxidative stress and improved cognitive impairment in Aβ_1–42_-induced AD mice, indicating that DG exerted potential beneficial effects on AD.

## Introduction

Alzheimer's disease (AD), with typical pathological abnormalities including amyloid plaques, neurofibrillary tangles and neuron death, is the most prevalent neurodegenerative disease [1]. Beta-Amyloid (Aβ) is the primary component of senile plaques and Aβ-induced oxidative stress and neuronal apoptosis play an important role in the pathogenesis of AD [2], [3], [4]. Persuasive evidence indicates that Aβ leads to the mitochondrial dysfunction partially by causing an imbalance of mitochondrial fission/fusion and impairing the mitochondrial biogenesis [5], [6], [7]. Moreover, Aβ was demonstrated to interact with Aβ-binding alcohol dehydrogenase (ABAD), which caused the release of reactive oxygen species (ROS), diminished cytochrome c activity and ATP depletion in AD patients and transgenic mice [8], [9], [10]. Thus, one promising preventive or therapeutic strategy for treatment of AD may be to attenuate or suppress Aβ-mediated oxidative stress and mitochondrial dysfunction.

The peroxisome proliferator-activated receptor gamma coactivator 1 (PGC-1) are a small family of transcriptional coactivators which play a critical role in the control of glucose, lipid, and energy metabolism [11]. There are three known isoforms of PGC-1: PGC-1α, PGC-1β and PGC-1-related coactivator. The physiological significance of PGC-1 in mitochondrial energy metabolism has been well demonstrated [12], [13]. Several groups including our studies have demonstrated that PGC-1α exerted neuroprotective effects in multiple neurological diseases [14], [15], [16], [17]. An intriguing finding in these studies was that PGC-1α null mice developed spongiform neurodegeneration in selective brain areas, which indicated the direct role of PGC-1α in the neurodegeneration [13]. PGC-1α was a direct target of cyclic AMP (cAMP) response element binding (CREB) *in vivo* and CREB-dependent gene expression played critical roles in the neuroplasticity associated with cognitive function [18].

Glycyrrhizin (GL), which is extracted in liquorice root, has a wide range of pharmacological actions including anti-virus, anti-allergenic and anti-immune-mediated cytotoxicity [19], [20], [21], . Diammonium glycyrrhizinate (DG), which is also extracted and purified from liquorices, is more stable, soluble and has more significant bioactivities than GL. DG has been used for treatment of hepatitis for many years in Asian countries because of its anti-inflammatory effect, resistance to biologic oxidation and membranous protection [23], [24]. This study demonstrated that DG suppressed Aβ_1–42_-induced oxidative stress and mitochondrial dysfunction partially via induction of PGC-1α and alleviated Aβ_1–42_-induced cognitive impairment, suggesting DG might be developed into a promising drug for treatment of AD.

## Materials and Methods

### Aβ_1–42_ induced AD mice model

The Aβ_1–42_ (Millipore, CA, USA) was dissolved in 1% NH_3_·H_2_O at a concentration of 1 µg/µl and incubated at 37°C for 5 days to allow for fibril formation. DG was purchased from Jiangsu Chia-Tai Tianqing Pharmacy Company. The male ICR mice (weight range: 15–20 g) were anesthetized and Aβ_1–42_ (4 µg, i.c.v) was injected to bilateral hippocampus by infusion cannulae. DG was co-injected intraperitoneally with Aβ_1–42_. The mice were randomly assigned into four groups: the normal mice with saline or DG (10 mg/kg/day, i.p. for 14 days), and Aβ_1–42_-induced AD mice with saline or DG (10 mg/kg/day, i.p. for 14 days). All animal experiments were approved by the Animal Care Committee in Nanjing University and performed according to institutional guidelines. We made every effort to minimize the number of mice used and their suffering.

### Cell culture and treatment

Primary cortical neurons were prepared from E15–17 mouse embryo. Cortexes were dissected and plated at 4×10^5^ cells/ml on poly-D-lysine-coated plates. Cells were maintained in Neurobasal media supplemented with B27 (Invitrogen, Carlsbad, California, USA) and 25 nM glutamine at 37°C in a humidified 5% CO_2_ incubator. The purity of neurons was over 95%. The cells at day 8 were incubated with 2 µM Aβ_1–42_ with DG or saline for 24 h.

HEK293T, BV-2 and RAW264.7 cells were obtained from American Type Culture Collection (ATCC) and maintained in DMEM containing 10% of heat-inactivated fetal bovine serum (FBS), 2 mmol/L of L-glutamine, 100 U/ml of penicillin, and 100 µg/ml of streptomycin at 37°C in a humidified 5% CO_2_ incubator.

### Plasmid construct and transient transfection

Small hairpin RNAs (shRNAs) were synthesized and subsequently cloned into pCMV-U6 vector using Bbsl and BglII (Fermentas Inc., USA). Five PGC-1α shRNAs sequences (shP1–shP5) were designed to target mouse PGC-1α gene. The plasmid expressing scrambled shRNA (sh-con) was used as a negative control. ShRNA sequences were as follows:

shP1: Forward: 5′-TTTGGCCATTGTTAAGACCGAGAATCTCGAGATTCTCGGTCTTAACAATGGCTTTTTG-3′, Reverse: 5′-GATCCAAAAAGCCATTGTTAAGACCGAGAATCTCGAGATTCTCGGTCTTAACAATGGC-3′;

shP2: Forward: 5′-TTTGCCCATTTGAGAACAAGACTATCTCGAGATAGTCTTGTTCTCAAATGGGTTTTTG-3′, Reverse: 5′-GATCCAAAAACCCATTTGAGAACAAGACTATCTCGAGATAGTCTTGTTCTCAAATGGG-3′;

shP3: Forward: 5′-TTTGCGGAGACTATTGAGCGAACCTTAACTCGAGTTAAGGTTCGCTCAATAGTCTTTTTTTG-3′, Reverse: 5′-GATCCAAAAAAAGACTATTGAGCGAACCTTAACTCGAGTTAAGGTTCGCTCAATAGTCTCCG-3′;

shP4: Forward: 5′-TTTGCGGTAACTATGCAGACCTAGATACCTCGAGGTATCTAGGTCTGCATAGTTATTTTTTG-3′, Reverse: 5′-GATCCAAAAAATAACTATGCAGACCTAGATACCTCGAGGTATCTAGGTCTGCATAGTTACCG-3′;

shP5: Forward: 5′-TTTGTCCAGTAAGCACACGTTTATTCTCGAGAATAAACGTGTGCTTACTGGATTTTTG-3′, Reverse: 5′-GATCCAAAAATCCAGTAAGCACACGTTTATTCTCGAGAATAAACGTGTGCTTACTGGA-3′.

sh-con: Forward: 5′-TTTGGCATTGCTTCTGTGTAAATTACTCGAGTAATTTACACAGAAGCAATGCTTTTTG-3′, Reverse: 5′-GATCCAAAAAGCATTGCTTCTGTGTAAATTACTCGAGTAATTTACACAGAAGCAATGC-3′.

The oligonucleotides were synthesized by Biocolor BioScience and Technology Company (Invitrogen, USA). shRNAs were transfected into neurons using Lipofectamine 2000 (Invitrogen, CA, USA) according to the manufacturer's instructions. Cells were harvested for RT-PCR and western blotting at 24 h after the transfection.

### Apoptotic assay by flow cytometry

Apoptosis was determined by Annexin V-FITC apoptosis detection kit (KeyGen Biotech, Nanjing, China). After treatment, the cells were rinsed with PBS twice, centrifuged at 600 g for 10 min and resuspended in 0.5 ml binding buffer containing 5 µl Annexin V and 5 µl propidium iodide (PI), and then incubated for 15 min at 37°C in the dark. The apoptotic rate was examined by flow cytometry.

### MTT assay

Cell viability was determined using the conventional MTT assay. After treatment, primary cortical neurons were treated with 0.5 mg/ml MTT for 4 h at 37°C. The formazan crystals were dissolved in 100 ml of DMSO and the absorbance was measured at 570 nm in a plate reader. Cell survival rates were expressed as percentages of the value of normal cells.

### LDH assay

LDH is the most widely used marker in cytotoxicity study. At the end of incubation, the supernatant was collected from plates and the LDH content was determined using an LDH assay kit according to the manufacturer's instructions (Nanjing Institute of Jianchen Biological Engineering, China). LDH cytotoxicity was calculated by the following formula: LDH cytotoxicity = (sample OD−blank OD)/(standard solution OD−blank standard solution OD )×2000.

### Measurement of mitochondrial membrane potential

Change of the mitochondrial transmembrane potential in neurons was quantified by JC-1 (Beyotime, Nanjing, China). Briefly, neuronal cells were centrifuged at 600 g for 10 min, and resuspended in 0.5 ml medium containing 5 µM JC-1. After 20 min of incubation at 37°C in the dark, the cells were washed with PBS twice and resuspended in 0.5 ml PBS. Samples were analyzed by flow cytometry.

### Measurement of intracellular ROS

To monitor intracellular accumulation of ROS, flow cytometry was used with commercial kit (Beyotime, Nanjing, China) according to the manufacturer's instructions. After treatment, the cells were harvested, rinsed with PBS twice, centrifuged at 600 g for 10 min, and then resuspended in 10 µM DCFH-DA solutions. After 20 min of incubation at 37°C, cells were washed with PBS twice and resuspended in 0.5 ml PBS. Samples were analyzed by flow cytometry.

### Measurement of 4-hydroxy-2- trans-nonenal (4-HNE)

The levels of 4-HNE from hippocampus and serum were measured by the ELISA kits (Genmed Scientifics Inc, USA) according to the manufacturer's instruction. Briefly, supernatant from hippocampus or serum were added into the 96-well plate coated with purified anti-4-HNE antibody, and then HRP-labeled 4-HNE antibody was added. The absorbance was measured at 450 nm and the concentration of 4-HNE was determined by comparing the O.D. of the sample to the standard curve.

### Cytochrome c detection

For measurement of cytochrome c release, the mitochondrial and cytosol fractions were prepared according to the manufacturer's instructions (Beyotime, Nanjing, China). Briefly, mice hippocampus were washed twice with cold PBS, resuspended in fresh cytosolic extract buffer and incubated for 30 min on ice with frequent tube tapping. Tissues were homogenized on ice, and then nuclei, unbroken cells, and cell debris were pelleted at 600 g for 10 min at 4°C. The supernatant was spun again at 13,000 g for 20 min at 4°C. The supernatant was carefully transferred and the final pellet was used as the mitochondrial fraction. The cytochrome c levels were determined using a monoclonal antibody to cytochrome c by western blotting as described below.

### Western blotting

Equal amounts of protein were separated by SDS-PAGE and electrophoretically transferred onto polyvinylidene fluoride membranes. Membranes were blocked with 5% non-fat dry milk for 1 h and incubated overnight at 4°C with rabbit anti-cleaved caspase-3 (1∶ 500, Bioworlde), rabbit anti-caspase-3 (1∶ 500, Bioworlde), rabbit anti-caspase-9 (1∶ 1,000, Cell signaling), rabbit anti-PGC-1α (1∶ 1000, Millipore), mouse anti-cytochrome c (1∶ 500, Abcam), or mouse anti-GAPDH (1∶ 5000, Bioworlde) antibody. GAPDH was used as a loading control. The proteins were detected with horseradish peroxidase-conjugated anti-rabbit or anti-mouse secondary antibodies and visualized with chemiluminescence reagents provided with the ECL kit (Amersham Pharmacia Biotech, Piscataway, NJ, USA) and exposure to film. The intensity of the blots was quantified with densitometry.

### Caspase-9 and -3 activity assay

Caspase-3 and caspase-9 activities of primary cortical neurons were measured by means of colorimetric assay kits (Keygen BioTech, Nanjing, China), according to the manufacturer's instructions. In brief, harvested cells were incubated with 50 µl lysis buffer on ice for 30 min, followed by centrifugation at 10,000 g for 1 min at 4°C. Then, cells were suspended in 50 µl 2×reaction buffer and 5 µl caspase-3 or caspase-9 substrate incubating for 4 h at 37°C. Later, the absorbance was read in a microplate reader at 400 nm.

### Real-time PCR and RT-PCR

Real-time PCR was performed as described previously [14]. Total RNA was extracted by using the Trizol reagent (Takara, Dalian, China) and was reverse-transcribed into cDNA using a PrimeScript RT reagent kit (Takara, Dalian, China) for Quantitative PCR (ABI 7500, USA) in the presence of a fluorescent dye (SYBR Green I; Takara). The relative abundance of mRNA was calculated after normalization to GAPDH mRNA. The primers are as follows:

PGC-1α: Forward: 5′-TGACACAACGCGGACAGAA-3′, Reverse: 5′-GGTAGGTGATGAAACCATAG-3′;

GAPDH: Forward: 5′-GCCAAGGCTGTGGGCAAGGT-3′, Reverse: 5′-TCTCCAGGCGGCACGTCAGA-3′.

The PCR products were analyzed on 1.5% agarose gels and visualized by ethidium bromide. The gel was visualized with UV-transilluminator and photographed.

### Luciferase reporter activity assays

The promoter regions of mouse PGC-1α (−3000 to 0 bp) were amplified using PCR, DNAs of primary cortical neurons as templates, and specific primers with MluI and BglII restriction enzyme (Fermentas Inc., USA) cut sites engineered on the ends (Forward: 5′-ATAAACGCGTAATGTGTGGCCGAACACACTGT-3′, Reverse: 5′-CGCCGAGATCTAAAGCTATTAAAAAGTAGGCT-3′) to facilitate directional cloning. The PCR products were cloned into the pGL3 basic in sense orientation (designated as p-PGC-3K). The truncated constructs were made using the following primers:

−3000–2500 bp (named as p-PGC-0.5K): Forward: 5′-ATAAACGCGTAATGTGTGGCCGAACAC-3′, Reverse: 5′-GTCGAGATCTCTTGTGTTTCTGCTGCTA-3′;

−3000–2000 bp (named as p-PGC-1K): Forward: 5′-ATAAACGCGTAATGTGTGGCCGAACAC-3′, Reverse: 5′-GTCGAGATCTTCTACTTTCCACACAGTC-3′;

−3000–1500 bp (named as p-PGC-1.5K): Forward: 5′-ATAAACGCGTAATGTGTGGCCGAACAC-3′, Reverse: 5′-CCGCCGAGATCTTCTGACTTTATATAGTC-3′;

−3000–1000 bp (named as p-PGC-2K): Forward: 5′-ATAAACGCGTAATGTGTGGCCGAACACACT-3′, Reverse: 5′-GCCGAGATCTTCCAACCCTAGTGCCTTG-3′;

−3000–500 bp (named as p-PGC-2.5K): Forward: 5′-ATAAACGCGTAATGTGTGGCCGAACACACT-3′, Reverse: 5′-GCCGAGATCTGATTTTCTTTCTCTCTCTCCT-3′;

−500–0 bp (named as p-PGC-500 bp): Forward: 5′-AAATAAACGCGTGGGGGTGTTGCCTTCAAAC-3′, Reverse: 5′-GCCCCGAGATCTAAAGCTATTAAAAAGTAGG-3′.

The sequence of −100–0 bp in the PGC-1α promoter (named as p-PGC-100 bp), CREB binding site mutation sequence (named as p-PGC-100 bp mutate) and deletion sequence (named as p-PGC-100 bp delete) were synthesized with MluI and BglII restriction enzyme cut sites as followers:

p-PGC-100 bp: Forward: 5′-CGCGTGAGGGCTGCCTTGGAGTGACGTCAGGAGTTTGTGCAGCAAGCTTGCACAGGAGAAGGGAGGCTGGGTGAGTGACAGCCCAGCCTACTTTTTAATAGCTTTA-3′, Reverse: 5′GATCTAAAGCTATTAAAAAGTAGGCTGGGCTGTCACTCACCCAGCCTCCCTTCTCCTGTGCAAGCTTGCTGCACAAACTCCTGACGTCACTCCAAGGCAGCCCTCA-3′;

p-PGC-100 bp mutate: Forward: 5′-CGCGTGAGGGCTGCCTTGGAGTGTGGTCAGGAGTTTGTGCAGCAAGCTTGCACAGGAGAAGGGAGGCTGGGTGAGTGACAGCCCAGCCTACTTTTTAATAGCTTTA-3′, Reverse: 5′-GATCTAAAGCTATTAAAAAGTAGGCTGGGCTGTCACTCACCCAGCCTCCCTTCTCCTGTGCAAGCTTGCTGCACAAACTCCTGACCACACTCCAAGGCAGCCCTCA-3′;

p-PGC-100 bp delete: Forward: 5′-CGCGTGAGGGCTGCCTTGGAGGGAGTTTGTGCAGCAAGCTTGCACAGGAGAAGGGAGGCTGGGTGAGTGACAGCCCAGCCTACTTTTTAATAGCTTTA-3′, Reverse: 5′-GATCTAAAGCTATTAAAAAGTAGGCTGGGCTGTCACTCACCCAGCCTCCCTTCTCCTGTGCAAGCTTGCTGCACAAACTCCCTCCAAGGCAGCCCTCA-3′.

All transfection experiments in this study were performed with Lipofectamine 2000 (Invitrogen) following the manufacturer's instructions. pPGCs and phRL-CMV *Renilla* were cotransfected to cells followed by DG treatment for 24 h. The Luciferase activity was assayed by using the Promega Bright-N-Glo system as previously described [25]. All data points were the averages of at least four independent transfections.

### Morris water maze test

The Morris water maze test was conducted as previously described [26]. Briefly, mice were trained to find a transparent Plexiglas platform in the pool placed 2 cm below the water surface in the middle of one quadrant. The position of the platform was unchanged during the training trials. Four time training trails per day were conducted for four consecutive days from 14 days after the injection. In each trial, the latency to escape on the platform was recorded for 1 min. Data of each mice behavior were collected by a video camera linked to a computer through an image analyzer. The total sum of latency and searching distance for the platform in four trials of each mouse was counted for all tested mice per group per day. At the end of the training period, mice were tested on a spatial probe trial in which the platform was removed from the pool, and each mouse was allowed to swim freely for 1 min. During the probe trial, the number of platform crossings was recorded. The recorded data were used to analyze mice performance.

### Statistical analysis

The data were expressed as means ± SEM and analyzed by SPSS12.0 statistical analytical software (SPSS, Chicago, IL, USA). Group differences in the escape latency, searching distance and swimming speed during the Morris water maze test were analyzed using two-way analysis of variance (ANOVA) with repeated measures followed by Bonferroni *post hoc* test with day and treatment as the sources of variation. Otherwise comparison between two groups was statistically evaluated by Student's t-test and multiple group comparisons were analyzed by one-way ANOVA followed by Tukey *post hoc* test. Values of *P*<0.05 were considered statistically significant.

## Results

### DG protects neurons from Aβ_1–42_-induced neurotoxicity *in vitro*

**Figure 1:**
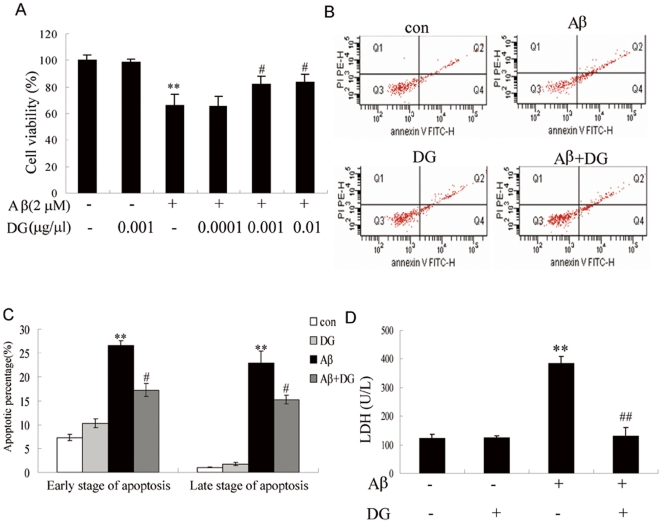
Protective effects of DG on Aβ_1–42_-induced cytotoxicity in primary cortical neurons. (A) Neurons were incubated with 2 µM Aβ_1–42_ and different concentrations of DG for 24 h, and the cell viability was estimated by the MTT assay. (B) Assessment of apoptosis in neurons incubated with 2 µM Aβ_1–42_ and 0.001 µg/µl DG for 24 h by Annexin V/PI double-staining. (C) Quantitative analysis of apoptotic cells. (D) LDH assay of neurons incubated with 2 µM Aβ_1–42_ and 0.001 µg/µl DG for 24 h. All data shown represent the mean± SEM of at least three independent experiments. ** *P*<0.01 for one-way ANOVA followed by Tukey *post hoc* test compared with control; # *P*<0.05 and ## *P*<0.01 for one-way ANOVA followed by Tukey *post hoc* test compared with Aβ_1–42_-treated, respectively.

To investigate whether DG could suppress the cellular toxicity induced by Aβ, the primary cortical neurons were incubated with Aβ_1–42_ (2 µM) and different concentrations of DG or saline. As expected, the viability of cortical neurons exposed to Aβ_1–42_ was reduced by 34.1% in comparison with the control group (*P*<0.01, Figure 1A) and DG significantly enhanced neuron viability (*P*<0.05, Figure 1A).

To further confirm the neuroprotection of DG on Aβ_1–42_-mediated toxicity, neuronal apoptosis was detected by Annexin V and PI staining. As shown in Figure 1B and 1C, cell apoptosis was demonstrated in Aβ_1–42_-treated neurons compared with the control, while significantly attenuated after treatment of DG. In addition, the inhibition of Aβ_1–42_-induced neuronal death in presence of DG was confirmed by the LDH assay (Figure 1D).

### DG decreases the oxidative damage induced by Aβ_1–42_

**Figure 2:**
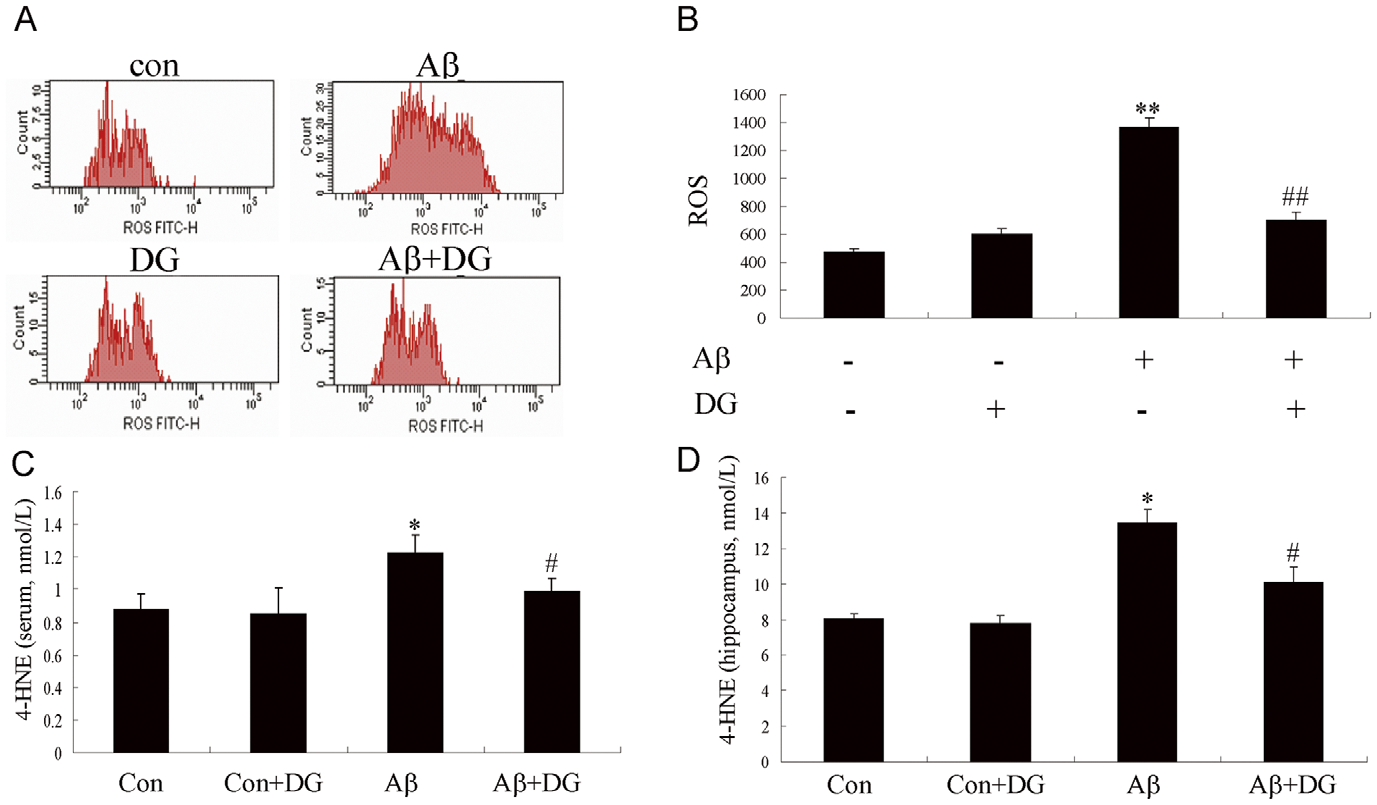
DG treatment decreased the oxidative stress induced by Aβ_1–42_. (A) Neuronal cells were treated with 2 µM Aβ_1–42_ and 0.001 µg/µl DG for 24 h and intracellular ROS was determined by flow cytometry. (B) Quantitative analysis of ROS. (C) The concentration of 4-HNE in the serum of normal and Aβ_1–42_-treated AD mice with saline or DG were determined by ELISA. (D) The concentration of 4-HNE in the hippocampus of normal mice with saline or DG, and Aβ_1–42_-induced AD mice with saline or DG were determined by ELISA. Results were shown as the mean± SEM and represented at least three independent experiments. * *P*<0.05 and ** *P*<0.01 for one-way ANOVA followed by Tukey *post hoc* test compared with control, respectively; # *P*<0.05 and ## *P*<0.01 for one-way ANOVA followed by Tukey *post hoc* test compared with Aβ_1–42_-treated, respectively. n = 5 mice per group.

Emerging evidence suggests that mitochondrial dysfunction and oxidative stress are involved in Aβ_1–42_-induced neurotoxicity. Thus, this study surmised that DG might be able to reduce Aβ_1–42_-mediated mitochondrial dysfunction and excessive production of ROS which was mainly produced by mitochondria. As shown in Figure 2A and 2B, exposure of cortical neurons to Aβ_1–42_ led to an increase in ROS production; whereas the effect was significantly decreased by treatment with DG (1365.67±67.52 vs. 705.67±51.87, *P*<0.01). To further investigate DG's ability to inhibit Aβ-induced oxidative stress, a marker of lipid peroxidation, 4-HNE was examined. As shown in Figure 2C and 2D, 4-HNE levels in the serum and hippocampus of Aβ_1–42_-induced AD mice were significantly increased by 40.4% and 67.3% compared to control mice respectively (*P*<0.05), while 4-HNE levels were reduced 24.2% and 33.2% in the serum and hippocampus after DG treatment respectively (*P*<0.05).

### DG prevents mitochondrial dysfunction mediated by Aβ_1–42_

**Figure 3:**
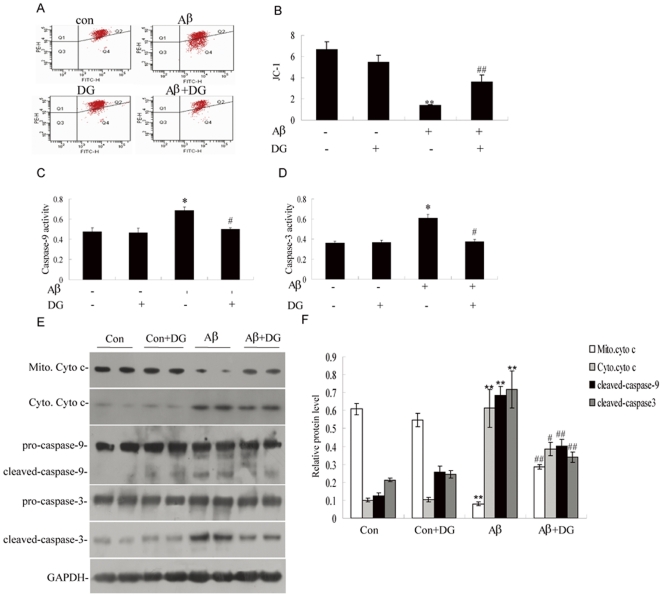
DG treatment rescued the mitochondrial dysfunction induced by Aβ_1–42_. (A) Neuronal cells were treated with 2 µM Aβ_1–42_ and 0.001 µg/µl DG for 24 h and mitochondrial membrane potential was measured by flow cytometry using JC-1. (B) Quantitative analysis of mitochondrial membrane potential. Neuronal cells were treated with 2 µM Aβ_1–42_ and 0.001 µg/µl DG for 24 h and Caspase-9 (C) and caspase-3 (D) activities were measured by means of colorimetric assay kits. (E) The expression of cytosolic, mitochondrial cytochrome c, caspase-9 and caspase-3 of hippocampus of normal mice with saline or DG, and Aβ_1–42_-induced AD mice with saline or DG was determined by western blotting. (F) Quantitative analysis of the relative protein levels of Figure 3E. Results were shown as the mean± SEM and represented at least three independent experiments. * *P*<0.05 and ** *P*<0.01 for one-way ANOVA followed by Tukey *post hoc* test compared with control, respectively; # *P*<0.05 and ## *P*<0.01 for one-way ANOVA followed by Tukey *post hoc* test compared with Aβ_1–42_-treated, respectively. n = 4 mice per group.

Mitochondrial membrane potential (Δψ) is widely recognized as an indicator of mitochondrial functionality, which is measured by JC-1, a cationic lipophilic fluorescent. The results showed that there was a significant loss of Δψ in neurons treated with Aβ_1–42_ (Figure 3A and 3B, *P*<0.01). However, the decrease of Δψ induced by Aβ_1–42_ was greatly alleviated after DG treatment (Figure 3A and 3B, *P*<0.01), indicating that DG protected mitochondrial against Aβ_1–42_-induced injury. Meanwhile, the activities of caspase -9 and caspase-3, were assessed. As shown in Figure 3C and 3D, the activities of caspase-9 and caspase-3 were significantly increased by 44.80% and 68.17% of the control group in Aβ_1–42_-treated neurons, while DG-treated neurons exhibited lower caspase-9 and caspase-3 activities compared to Aβ_1–42_-treated neurons (*P*<0.05).

To further explore the role of DG against mitochondrial dysfunction induced by Aβ *in vivo*, the release of cytochrome c from the mitochondrial membrane as well as the subsequent activation of caspase-9 and caspase-3 was investigated by western blotting. The levels of cytosolic cytochrome c expression in Aβ_1–42_-induced AD mice were significantly increased, which were significantly reversed by the treatment with DG (Figure 3E and 3F). In addition, DG could inhibit the activation of caspase-9 and caspase-3 in Aβ_1–42_-induced AD mice (Figure 3E and 3F).

### PGC-1α may be involved in DG -afforded neuroprotection against Aβ_1–42_

**Figure 4:**
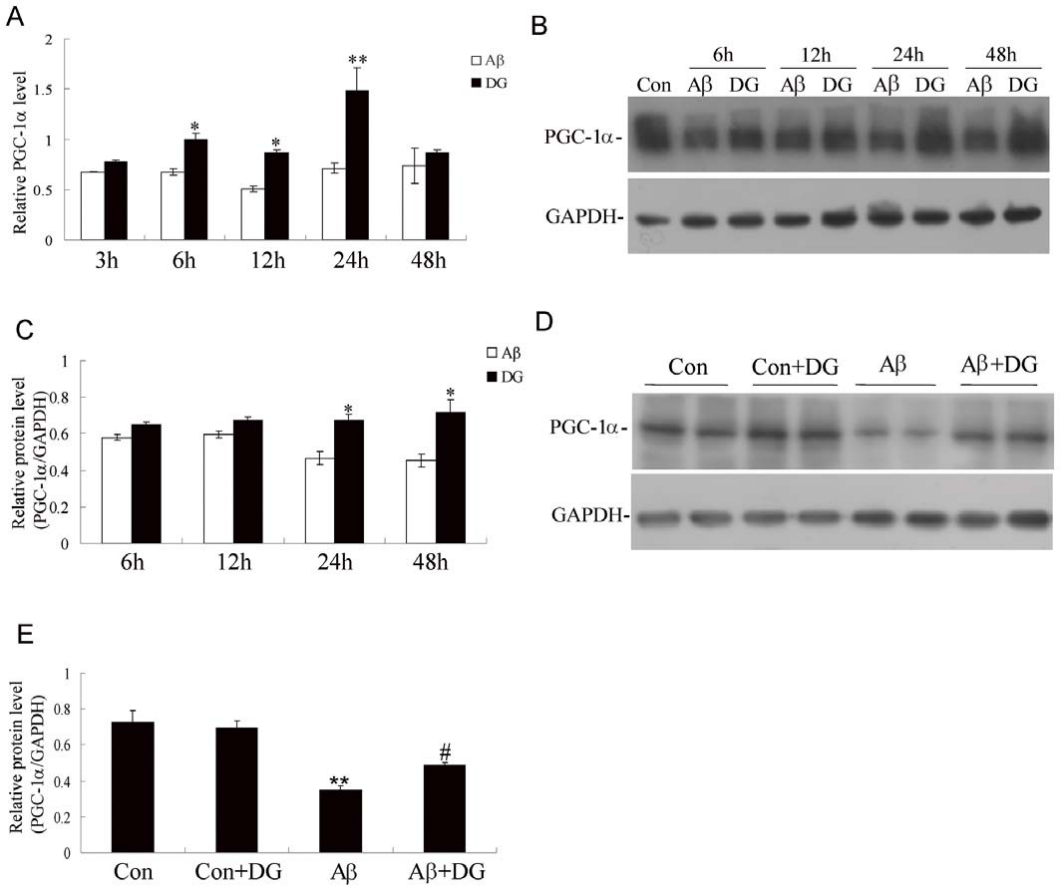
DG treatment significantly increased the expression of PGC-1α *in vitro and in vivo*. Neurons were treated with 2 µM Aβ_1–42_ and 0.001 µg/µl DG for the indicated time points, and the mRNA (A) and protein expression (B) of PGC-1α were analyzed by Real-time PCR and western blotting respectively. (C) Quantitative analysis of the relative protein levels of PGC-1α. The relative RNA or protein levels of control neurons were considered 1. Results were shown as the mean± SEM and represented at least three independent experiments. * *P*<0.05 and ** *P*<0.01 for Student's *t*-test compared with Aβ_1–42_-treated, respectively. (D) The expression of PGC-1α of hippocampus of normal mice with saline or DG, and Aβ_1–42_-induced AD mice with saline or DG was determined by western blotting. (E) Quantitative analysis of the relative protein levels of PGC-1α. Results were shown as the mean± SEM and represented at least three independent experiments. ** *P*<0.01 for one-way ANOVA followed by Tukey *post hoc* test compared with control mice; # *P*<0.05 for one-way ANOVA followed by Tukey *post hoc* test compared with Aβ_1–42_-treated AD mice. n = 4 mice per group.

**Figure 5:**
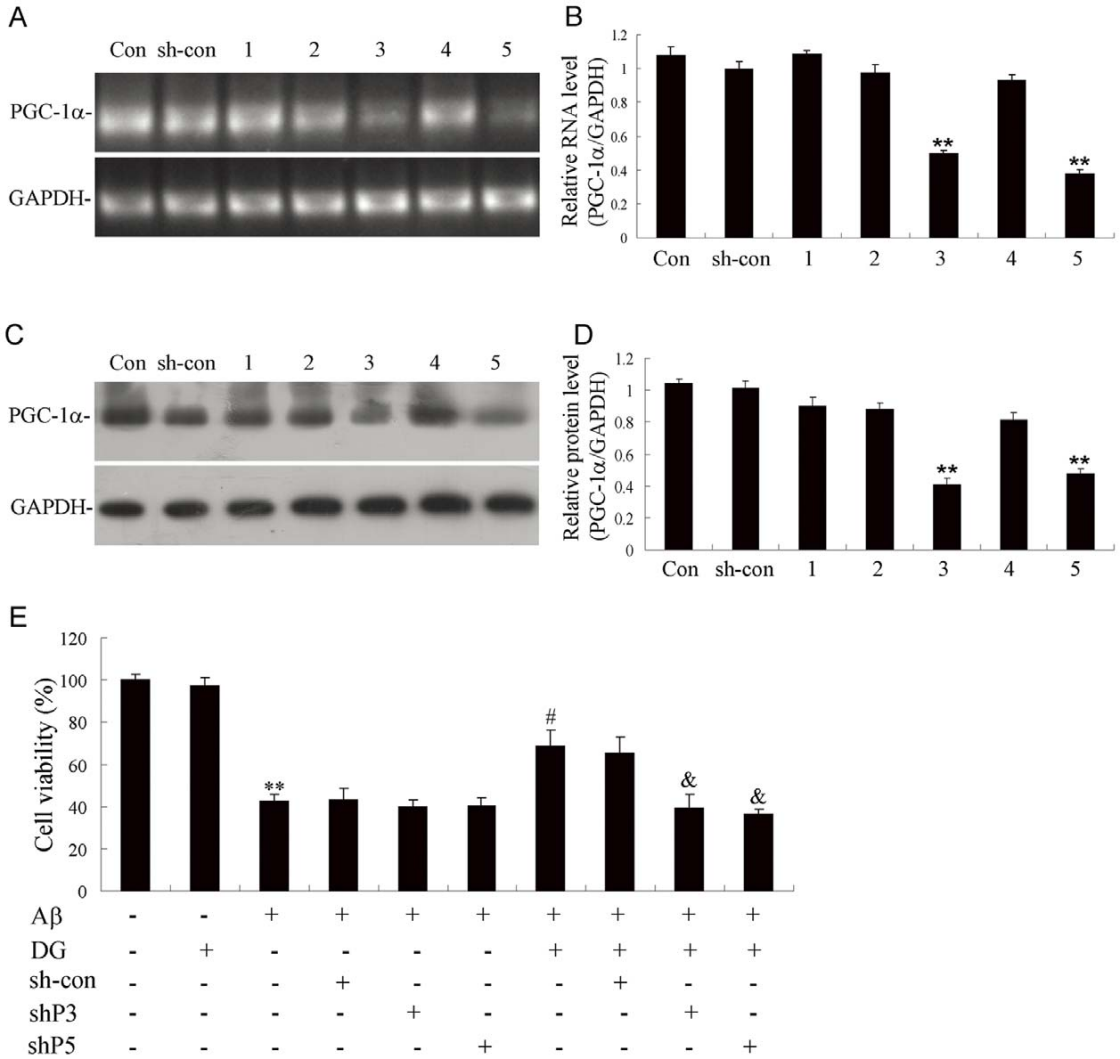
Inhibition of PGC-1α could partially block the neuroprotective effects of DG. Five shRNAs targeted to PGC-1α were constructed and the efficacy was confirmed by RT-PCR (A) and western blotting (B) in primary cortical neurons. (C) Quantitative analysis of the relative RNA levels of PGC-1α. (D) Quantitative analysis of the relative protein levels of PGC-1α. (E) Neuronal cells were transfected by shP3 or shP5, 6 h after which cells were treated with 2 µM Aβ_1–42_ and 0.001 µg/µl DG for another 24 h. Cell viability was estimated by the MTT assay. ** *P*<0.01 for one-way ANOVA followed by Tukey *post hoc* test compared with control; # *P*<0.05 for one-way ANOVA followed by Tukey *post hoc* test compared with Aβ_1–42_; & *P*<0.05 for one-way ANOVA followed by Tukey *post hoc* test compared with Aβ_1–42_+DG.

Next, whether the neuroprotection of DG against Aβ_1–42_-induced neurotoxicity was related to the expression of PGC-1α in neurons was addressed. The results indicated that DG markedly increased the mRNA expression of PGC-1α at 3 h (1.14±0.05-fold), 6 h (1.47±0.08-fold), 12 h (1.70±0.11-fold), 24 h (2.11±0.48-fold) and 48 h (1.24±0.38-fold) (Figure 4A), with similar protein expression pattern (Figure 4B and 4C). PGC-1α was also down-regulated in Aβ_1–42_-induced AD mice, while DG treatment could significantly increase its expression (Figure 4D and 4E). To demonstrate whether PGC-1α contributed to the neuroprotection of DG, endogenous PGC-1α was knocked down by shRNAs (Figure 5A to 5D) and the results of MTT revealed that PGC1α-shRNA could partially block neuroprotective effects by DG in Aβ_1–42_-treated neurons (Figure 5E).

**Figure 6:**
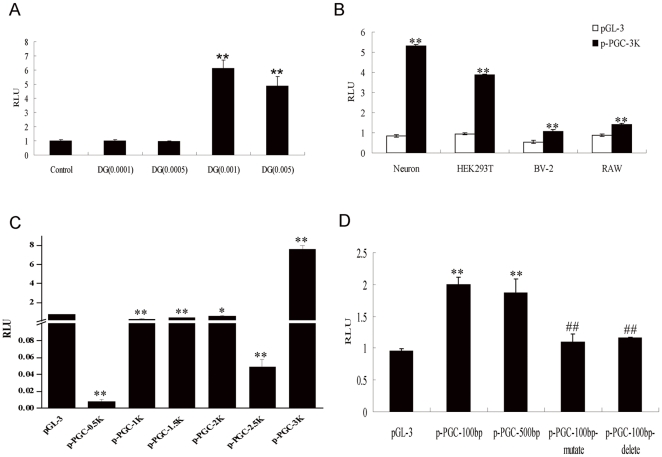
DG might induce the transcriptional activity of PGC-1α through CREB. (A) pPGC-3K and phRL-CMV *Renilla* were cotransfected to neurons followed by DG treatment at the indicated concentration and the luciferase activity was assayed at 24 h. Results are shown as the mean± SEM and represent at least four independent experiments. ** *P*<0.01 for one-way ANOVA followed by Tukey *post hoc* test compared with control. (B) pPGC-3K and phRL-CMV *Renilla* were cotransfected to neurons, HEK293T, BV-2, or RAW264.7 cells followed by DG treatment (0.001 µg/µl) and the luciferase activity was assayed at 24 h. ** *P*<0.01 for Student's *t*-test compared with pGL-3. (C) pPGCs and phRL-CMV *Renilla* were cotransfected to HEK293T cells followed by DG treatment (0.001 µg/µl) and the luciferase activity was assayed at 24 h. (D) pPGC-100 bp or mutated pPGC-100 bp or deleted pPGC-100 bp were cotransfected to HEK293T cells with phRL-CMV *Renilla* followed by DG treatment (0.001 µg/µl) and the luciferase activity was assayed at 24 h. Results are shown as the mean± SEM and represent at least four independent experiments. * *P*<0.05 and ** *P*<0.01 for one-way ANOVA followed by Tukey *post hoc* test compared with pGL-3, respectively; ## *P*<0.01 for one-way ANOVA followed by Tukey *post hoc* test compared with pPGC-100 bp.

To explore whether DG could induce the transcriptional activity of PGC-1α, p-PGC-3K and five truncated plasmids were constructed. As shown in Figure 6A, DG treatment significantly up-regulated the transcriptional activity of PGC-1α by 6.23-fold at a concentration of 0.001 µg/µl, and 5.13-fold at a concentration of 0.005 µg/µl in neurons (*P*<0.01). Also DG treatment significantly up-regulated the transcriptional activity of PGC-1α by 4.08-fold in HEK293T cells, 1.98-fold in BV-2 cells and 1.62-fold in RAW264.7 cells at a concentration of 0.001 µg/µl, indicating that induction of transcriptional activity of PGC-1α by DG may not be cell type specific (Figure 6B). Interestingly, DG significantly downregulated the transcriptional activity of p-PGC-2.5K while increasing the transcriptional activity of p-PGC-3K (*P*<0.01), which indicated that −500–0 bp in the promoter of PGC-1α might be involved in the protective effects of DG (Figure 6C). To explore whether the CREB binding sequence (−84–77 bp) was essential for the DG-induced transcriptional activity of PGC-1α, −100–0 bp of the PGC-1α promoter and the CREB binding sites mutated/deleted sequences were constructed. As shown in Figure 6D, mutation or deletion of the CREB binding sequence completely abolished the induction of the transcriptional activity of PGC-1α, which suggested that CREB might play an important role in the DG-induced transcriptional activity of PGC-1α.

### DG improves cognitive impairment in Aβ_1–42_-induced AD mice

**Figure 7:**
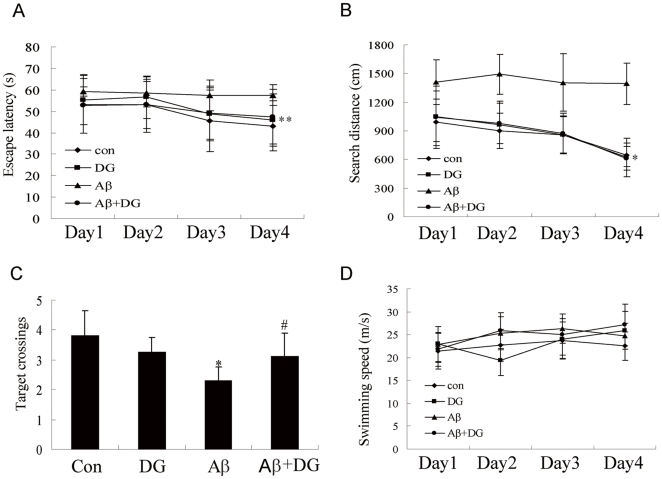
DG treatment (10 mg/kg/day, i.p. for 14 days) attenuated learning and memory impairment in Aβ_1–42_-induced AD mice. (A) Escape latency for escape to a submerged platform in the training trials. (B) Searching distance for escape to a submerged platform in the training trials. * *P*<0.05 and ** *P*<0.01 for two-way ANOVA with repeated measures followed by Bonferroni *post hoc* test compared with Aβ_1–42_-treated, respectively. (C) 24 h after the training trials platform crossing times were recorded. * *P*<0.05 for one-way ANOVA followed by Tukey *post hoc* test compared with control; # *P*<0.05 for one-way ANOVA followed by Tukey *post hoc* test compared with Aβ_1–42_-treated. (D) Swimming speed in the training trials. con: normal mice; DG: normal mice with DG (10 mg/kg/day, i.p. for 14 days); Aβ: Aβ_1–42_-induced AD mice; Aβ+DG: Aβ_1–42_-induced AD mice with DG (10 mg/kg/day, i.p. for 14 days). n = 10 mice per group.

To explore whether DG could improve cognitive impairment in Aβ_1–42_-induced AD mice, Morris water maze test was employed. Escape latency reflects the ability of learning and remembering the relationships between multiple distal cues and the platform location to escape the water, which is a hippocampus-dependent task. As shown in Figure 7A, DG could decrease the mean latency reaching to the submerged platform of AD mice (two-way ANOVA with repeated measures; groups: F(3, 31) = 6.123, *P* = 0.002; days: F(3, 93) = 3.620, *P* = 0.016; group x day: F(9, 93) = 0.915, *P* = 0.516). In addition, the search distance was also significantly decreased by DG treatment compared to AD mice (two-way ANOVA with repeated measures; groups: F(3, 17) = 41.688, *P* = 0.014; days: F(3, 51) = 11.536, *P* = 0.01; group x day: F(9, 51) = 1.942, *P* = 0.067, Figure 7B). On the fifth day, the platform was removed and the probe trail was conducted. AD mice had fewer times crossing the previous platform position than the normal mice, while those under the treatment of DG significantly improved their performance (*P*<0.05, Figure 7C). No speed differences appeared among these four groups (Figure 7D). It suggested that DG could alleviate the deficits of spatial learning and memory in Aβ_1–42_-induced AD mice.

## Discussion

In the Aβ_1–42_-induced AD model *in vitro* and *in vivo*, this study for the first time shows: 1) DG exerts neuroprotective effects and improves cognitive impairment; 2) DG rescues mitochondrial dysfunction and inhibits oxidative stress; and 3) DG increases the expression of PGC-1α, which might contribute to the neuroprotection of DG.

Mitochondrial dysfunction is a hallmark of Aβ_1–42_-induced neuronal toxicity, and is considered as an early event in AD pathology. Several evidences indicated that Aβ triggered mitochondrial dysfunction through a number of pathways such as increase of ROS, interaction with ABAD, impaired mitochondrial biogenesis, and alteration of mitochondrial dynamics [5], [6], [7], [10]. DG is the salt form of glycyrrhizin, a major active constituent isolated from licorice. Licorice and glycyrrhizin have anti-oxygenic and anti-inflammatory action in bile acid-induced apoptosis and necrosis [22] [27], [28]. The route of administration of DG was selected because of good bioavailability reported previously [29], and our results had shown that DG did not attenuate cognitive impairment in AD mice at high dose (50–100 mg/kg) (data not shown). This study indicated that DG could decrease the accumulation of ROS, rescue the mitochondrial membrane potential loss and activation of caspase-9 and caspase-3 in Aβ_1–42_-treated neurons. In addition, DG decreased lipid peroxidation and release of cytochrome c from the mitochondria, and the activation of caspase-9 and caspase-3 in Aβ_1–42_-induced AD mice. Furthermore, this anti-oxidation function of DG could refrain neurotoxicity mediated by Aβ_1–42_, that is, increased cell viability, decreased apoptosis and LDH release in Aβ_1–42_-treated neurons.

Regardless of the possible mechanisms of DG restraining oxidative stress, it is clear that PGC-1α is a major regulator of mitochondrial biogenesis and is protective against oxidative damage [30]. In addition, PGC-1, which could bind to and activate many other transcription factors, played an essential role in physiological signaling transduction and gene expression [31]. However, the role of PGC-1α in the neurological diseases was not extensive studied until recently. PGC-1α null mice were much more sensitive to the neurodegenerative effects of MPTP and kainic acid, which were oxidative stressors. Increasing PGC-1α levels dramatically protected neurons from oxidative-stressor-mediated death [17]. Resveratrol was an ideal compound for treating neurodegenerative diseases by increased the activity of numerous proteins, including PGC-1α [32]. Activation or overexpression of PGC-1α could be used to compensate for neuronal mitochondrial loss [16]. Expression levels of PGC-1α were significantly decreased in both AD hippocampus and M17 cells stably expressing human Swedish mutation APP695 [5] . Consistent with these reports, our data showed that DG increased the expression of PGC-1α and that knocking down PGC-1α by shRNAs could block the neuroprotection of DG, suggesting that PGC-1α contributed to the neuroprotective effect of DG in Aβ_1–42_-treated neurons.

It is intriguing that DG treatment increased the expression of PGC-1α in Aβ_1–42_-treated neurons and CREB might play an important role in induction of the transcriptional activity of PGC-1α. PGC-1α was a direct target of CREB induction of gluconeogenesis *in vivo* [33]. CREB was also essential for long-lasting changes in synaptic plasticity that mediates the conversion of short-term memory to long-term memory. Aβ altered hippocampal-dependent synaptic plasticity and memory storage and mediated synapse loss through the CREB signaling pathway, which suggested a crucial role of CREB signaling in cognitive dysfunction [34]. Therefore, further studies are needed to demonstrate the exact role of CREB to the transcriptional activity of PGC-1α and neuroprotective effects of DG.

Taken together, DG exerted neuroprotective effects against Aβ_1–42_-induced toxicity *in vitro* and *in vivo*. DG significantly increased the viability of Aβ_1–42_-treated neurons by inhibiting oxidative stress and reversing mitochondrial dysfunction. Furthermore, PGC-1α upregulated by DG treatment might play an important role against Aβ_1–42_-induced neurotoxicity. Findings of current study revealed new function and mechanism of DG on neurotoxicity induced by Aβ_1–42_, suggesting that DG may be developed into a new drug for treatment of AD.
